# Access to Affordable Health: A Care Delivery Model of GNRC Hospitals in North-Eastern India

**DOI:** 10.5334/ijic.7587

**Published:** 2024-02-26

**Authors:** Nomal Chandra Borah, Priyanka Borah, Satabdee Borah, Madhurjya Borah, Purabi Sarkar

**Affiliations:** 1 Centre for Affordable Health Mission, GNRC Hospitals, Dispur-781006, Assam, India; 2 Centre for Affordable Health Mission, GNRC Hospitals, Sixmile-781022, Assam, India; 3 Centre for Affordable Health Mission, GNRC Hospitals, North Guwahati-781039, Assam, India; 4 Department of Research and Analytics, GNRC Hospitals, Dispur, Assam-781006, India

**Keywords:** affordable care, healthcare delivery, integrated care, public health, population health

## Abstract

**Introduction:**

The healthcare delivery system of Assam faces several challenges to provide affordable, accessible and quality care services. GNRC (Guwahati Neurological Research Center) is the first super-speciality hospital to address many of these gaps by delivering integrated affordable healthcare services to the populations of Assam and other parts of North-eastern India.

**Description & Discussion:**

This paper describes the implementation of a care delivery model which provides integrated care delivery services through linking hospitals to primary healthcare services, including preventive, promotive, and curative care, along with delivering easily accessible and affordable care to the people of Assam and other parts of North-eastern India.

**Conclusion:**

The proposed model is the first innovative approach from North-eastern India, Assam, to deliver affordable, accessible and patient-centric hospital led community-based preventive, promotive, and primary, secondary, and tertiary hospital-based care. It is anticipated that GNRC’s “Affordable Health Mission” will help redesign and integrate the way primary, secondary and tertiary healthcare is delivered to the population of Assam in helping patients manage their own health and reduce the numbers that needs to be admitted to secondary care and tertiary care by improving patients’ independence and well-being as well as dramatically reducing the cost to the overall health system.

## Introduction

The health of populations is changing globally, which is critical for human dignity and essential for socio-economic growth [1]. Health is not merely a basic requirement for an individual but it is also an integral component of human capital for the nation. Due to the accelerated rise in chronic non-communicable ailments, like hypertension, diabetes, stroke, etc., which dominate communicable diseases such as Japanese encephalitis, dengue, and typhoid, etc. the total disease burden of the country, that requires effective and efficient healthcare delivery systems to overcome it [2]. So, healthcare providers around the world are seeking to manage the rising burden of chronic conditions for both growing and aging populations as well as greater expectations of health services. Access to appropriate, adequate, and affordable health care is the legitimate entitlement of every Indian citizen during his or her life. Though several pieces of evidence from different countries have shown that fragmentation of care adversely impacts quality, cost, health status, and patient satisfaction, integrated care can diminish many of these problems [3,4,5,6,7,8,9,10]. So, there is a need for an essential change in the healthcare system to promote equity, efficiency, effectiveness, and accountability in the healthcare delivery system. This can be done through the establishment of an integrated affordable care delivery model.

The healthcare delivery system of Assam also faces several challenges such as poor health-seeking behaviour combined with poor health awareness and poor penetration of healthcare insurance by private sectors, to provide affordable, accessible and quality care services. Moreover, in Assam, there is a huge out-of-pocket expense in healthcare, and fewer numbers of doctors, nurses, and hospital beds as compared to the national average. While states incur a high OOPE (54.2%) [11] with 6% of the families face catastrophic health expenditures [11], the insurance coverage in the state appears to be very low (2.6%) [11]. So, due to catastrophic healthcare expenditure and lack of affordable quality healthcare over 55 million people slide into poverty every year in India alone, which neutralizes the gains of rising income and various government initiatives aimed to reduce poverty. Therefore, government of India aims to provide quality healthcare to its citizens at an affordable cost through various initiatives and schemes. Guwahati Neurological Research Center’s (GNRC’s) “Affordable Health Mission (AHM)” is one such noble initiative in Assam to facilitate affordable and accessible healthcare for all.

Therefore, with the aim to provide quality and affordable care GNRC Hospitals, a private sector super-speciality tertiary-care hospital in Assam has developed a low-cost, affordable, and innovative care delivery model through vertical integration of hospital based secondary and tertiary services with out-patient primary and community based preventive care, named as “Affordable Health Mission”. The main objective of this mission is to ensure access to quality and affordable healthcare services for every person in the society irrespective of their financial background. It is currently active in all of the 35 districts of Assam and 2 districts in West Bengal and aims to cover all the districts in India in the next few years.

## Current scenario of the health sector in Assam

Assam the land of blue hills, valleys, mighty river the Brahmaputra, lies in the northeastern corner of India with a geographical area of 78,438 square km. Despite the richness of natural endowment, the region is one of the most backward areas of the country [12]. As per the 2011 census, the state has a population of 31 million, mostly with 86% rural population and one third of the state is poor according to World Bank (32%) [13]. In Assam, the incidence of poverty remains higher than the national average, with poverty levels being very high in some parts of the state [14]. Assam has the highest maternal mortality (237 per 100,000 live births) [15], the second-highest infant mortality (44 per 1000 live births) [15], and the lowest life expectancy at birth in the country (63.9 years) [15]. The state is also grappling with a very high prevalence of non-communicable diseases (NCDs), with more than half the deaths caused by NCDs, especially cardiovascular diseases and cancers [16].

## Health Care delivery system in India

Indian healthcare delivery system is inclusive of both public and private service providers that are organized into primary, secondary, and tertiary care levels [17]. However, most of the private healthcare providers are concentrated in urban India, providing secondary and tertiary care healthcare services [18].

## Public healthcare providers

The central government, the state governments, and local bodies [19,20] provide public healthcare facilities in India. They provide preventive, promotive, and curative care though the focus is to give maternal and child health care. Through ASHA and Anganwadi health workers, the public healthcare system is providing affordable and accessible healthcare to community people. Despite the low cost of health care in public health facilities, poor households incur a high catastrophic health spending (CHS) and bear a higher burden of diseases [21,22,23,24]. It is due to the high (54.2%) OOPE of the states [11] where 6% of the families face catastrophic health expenditures [11], and the insurance coverage in the state appears to be very low (2.6%) [11]. Moreover, almost 64% of health expenditure is on medicines, followed by diagnostics and transportation (38%) which is one of the highest in the country (compared to a national average of 16%) [11]. Several shreds of evidence show that despite an increase in insurance coverage financial risk protection has not been reduced [25,26,27]. Several publicly funded health insurance (PFHI) schemes, such as Rashtriya Swasthya Bima Yojana (RSBY), the Vajpayee Arogyashree Scheme (VAS), Rajiv Arogya Shree (RAS), and Comprehensive Health Insurance (CHIS) have been operational in India since 2008. These schemes have been impactful in increasing healthcare utilization in terms of outpatient and in-patient care in both rural and urban areas. However, evidence related to financial risk protection was mixed and inconclusive [28]. Moreover, the public system suffers from challenges of shortfall and supply-side constraints such as the availability of trained medical personnel and the non-availability of advanced diagnostic tools and technology. Additionally, since their focus is mainly on maternal and child care, a majority of the health needs of the community go unmet.

## Private Healthcare providers

The private sector is widely variable in quality and is diverse, with small, medium and large size hospitals along with individual practices, diagnostics labs, chemists, and traditional healers. In India, though private healthcare provides timely and quality treatment as compared to public sector healthcare facilities it comes with a price to pay, which is much more expensive than those in the public sector. Mostly primary care is provided at clinical levels for out-patients. There are several drawbacks of the private sector healthcare system such as they are mostly focused on curative care, and no focus is given on promotive and preventive care. Out-of-pocket expenses are too high in private sectors which leaves, more than 80 percent of the population outside the purview of private healthcare due to affordability issues. Till now there is no attempt to bring down the cost in a systematic way. Government-sponsored schemes such as Ayushman Bharat are also unattractive due to the unsustainable pricing of healthcare facilities.

## Description of the care practice

Intending to make quality healthcare available to everyone at affordable rates, GNRC’s “Affordable Health Mission” is a unique and ambitious model to minimize out-of-pocket medical expenses. The primary objective is to provide preventive, promotive, and curative care and create widespread awareness – through education and advocacy – about the adverse impact of lack of access to affordable healthcare on individuals, societies, and economies.

## Overview of GNRC hospitals

GNRC (formerly known as Guwahati Neurological Research Center) the first super-speciality healthcare centre in North Eastern India, was established in 1987 to provide services to all populations of Assam and outreach to underserved communities. After running GNRC as a super-specialty hospital for years, the organization went through a process of revisiting its strategy to formulate a model that enabled access to quality, affordable healthcare for the majority of the population. GNRC conducted numerous surveys and interviews with members of the local communities in an attempt to understand the challenges they face in accessing affordable quality healthcare. These conversations led to the formulation of an operating and service delivery model called “Affordable Heath Mission”. Followed by this, in the year 2012, GNRC started its community outreach program which is known as Medireach, and then the community health worker program known as Swasthya Mitra. Thus began GNRC’s new focus on delivering “affordable care” to Assam’s population by improving efficiencies of integrating primary, secondary, and tertiary care, along with preventive and promotive care. The main focus of all GNRC’s innovations was to increase cost efficiencies in each component of care delivery. Presently, GNRC operates five hospitals, providing quality, affordable care to over 250,000 out-patients and 25,000 in-patients annually. Together, the five campuses account for over 750 beds which offer services across 21 specialties with state-of-the-art diagnostic facilities and fully stocked pharmacies. Some of the areas GNRC focuses on detection and early intervention and management of Road traffic accidents (RTA), Stroke, Coronary artery disease (CAD), chronic kidney disease, etc. As compared to other private players, GNRC provides services with price differences of more than 50% in some cases; free OPD charges, and free of cost emergency care for the first 24 hours.

## Components of the “Affordable Health Mission” model

The “AHM” mainly works on five principles- Disease Prevention, Awareness, Disruptive innovation for an affordable cost, Alignment with government schemes, and Economic Empowerment to ensure access to quality healthcare for all with its various initiatives.

- **Disease Prevention:** We have realized that about 60 to 70% of the diseases common people often suffer could be prevented if care can be given at times to the patients. Therefore, it is essential to keep them healthy and disease-free rather than treating them when they become sick. At times when the disease becomes complicated, due to delayed care or lack of care, then the hospitals cannot achieve the desired levels of cure, even when the patient is admitted. Again, after patients are discharged from the hospital, they are on their own, without continuous disease management which puts patients in a repetitive cycle of care or waiting till it is too late. Therefore, prevention plays a significant role in the making of a healthy society.
- **Awareness**: Most people arrive late for treatment in the hospital due to ignorance, financial problem, and poor health-seeking behaviour. As a result, treatment gets longer and more complicated, and the treatment outcome also becomes poor. A lack of awareness about diseases, their symptoms, and their consequences leads to a tendency of delaying medical help. So, it is of the utmost importance to make people aware of health-seeking behavior and not to neglect one’s treatment at the beginning itself and take initiative for their treatment that will give faster, better, and cost-effective treatment.
- **Disruptive innovation for Affordable cost:** For quality care, apart from accessibility, affordability is a matter of global concern for low-income and vulnerable people in the society. Mostly, common people refrain from healthcare facilities because of their unaffordability to access hospitals, due to the rising costs. Therefore, through disruptive innovation GNRC is trying to bring down costs and make them affordable to a large number of people. This type of innovation will help underprivileged people to get better quality treatment at an affordable price.
- **Alignment with government schemes:** To provide quality healthcare services to the patients, GNRC has aligned with two flagship schemes, Ayushman Bharat (PM-JAY) and Atal Amrit Abhiyan of the government of India and the Government of Assam respectively and till now we have been able to serve a sizeable number of beneficiaries of these schemes at GNRC.
- **Economic Empowerment:** We have realized that poverty, ignorance, and ill health are interlinked. So, unless we break the cycle of poverty, and ill health people will become sick again and again and this will continue forever. So, to address this problem we are working for the economic empowerment of low-income people for enhancing their income through some schemes. So, finding out innovative ways to economically empower these people is equally crucial so that the basic minimum needs of life and health are ensured.

## Facilities provided under “Affordable Health Mission” (AHM)

**Figure 1:**
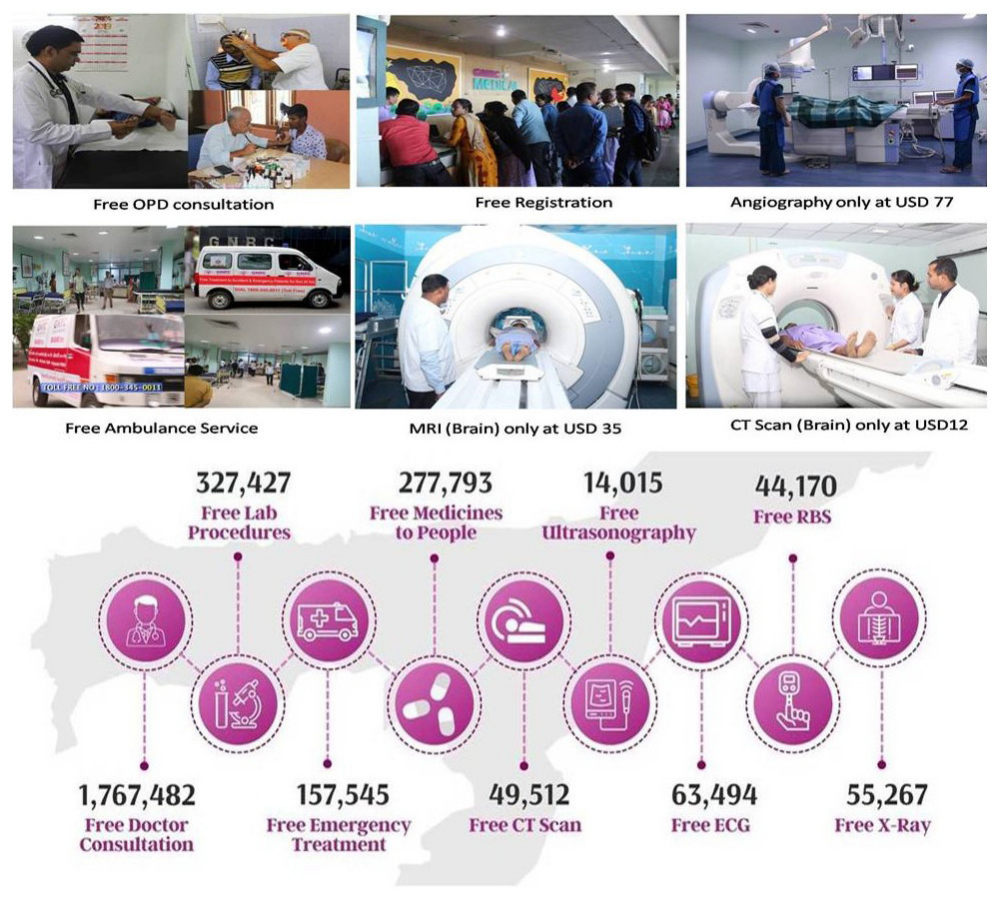
Number of lives touched under the affordable health mission: 1.7 million people (from March 2012 to till date).

The formal launch of the “Affordable Health Mission” (AHM) was done in the year 2017 to provide low-cost, affordable, and quality healthcare to all sections of society irrespective of their financial background. The unique and ambitious model of the GNRC’s Affordable Health Mission thrives continuously at the service of people with its unique and standalone initiatives. The following are the activities carried out under the “Affordable Health Mission” (Figure 1).

1. Free Ambulance Services
2. Free OPD Consultation
3. Nominal Registration charges
4. Free Treatment to Accident & Emergency patients for first the 24 Hours
5. Same day OPD to Prescription Services
6. Free Transport Services
7. Reduced Cost of Diagnostic Services
8. Reduced Cost of Surgeries

## GNRC’s Interventions aimed at Integrated Care

**Figure 2:**
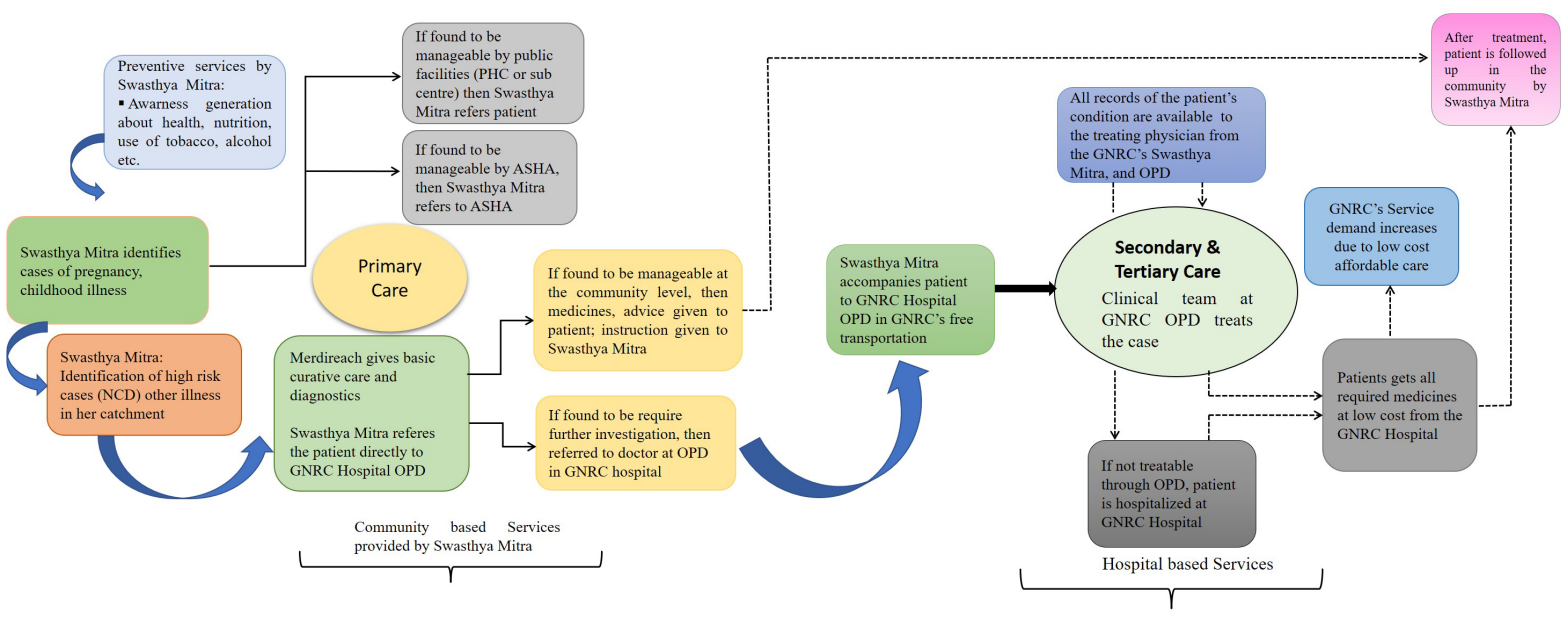
Pictorial representation of integrated care delivery model of GNRC hospitals.

GNRC hospitals have undertaken several interventions such as building low-cost hospital infrastructure, involving the catchment community in the supply chain and creating a sense of community ownership, training schools for nurses and specialists. The main focus of this case study is on GNRC’s interventions in leading affordable integrated care (Figure 2).

### Integrated care linking hospitals with primary care

Integration of GNRC’s hospital-based secondary and tertiary care with comprehensive primary care is the fundamental change in our delivery model. As per GNRC’s internal analysis, almost 45% of hospitalizations could be prevented if timely care can be given to the patients, especially for non-communicable conditions. Usually, patients are admitted in hospital in their advanced stage of the disease due to lack of care or delayed care therefore, hospitals cannot achieve the desired levels of cure. Moreover, once these patients are discharged, (in cases of a fragmented system), the patients are on their own, without continuous disease management which put them in the same cycle of foregone care or waiting till it is too late. The internal data also revealed that almost 85% of the cases that were in the GNRC Hospitals could have been managed at a primary care level. The hospitals were crowded with fairly simple and preventable causes, which make inefficient use of expensive resources and scarce specialists’ time. This had two direct impacts – first patients were spending money wastefully, and second, the efficiency of the hospitals was lower.

## Some of the key innovations to GNRC’s model

### Medireach

**Figure 3:**
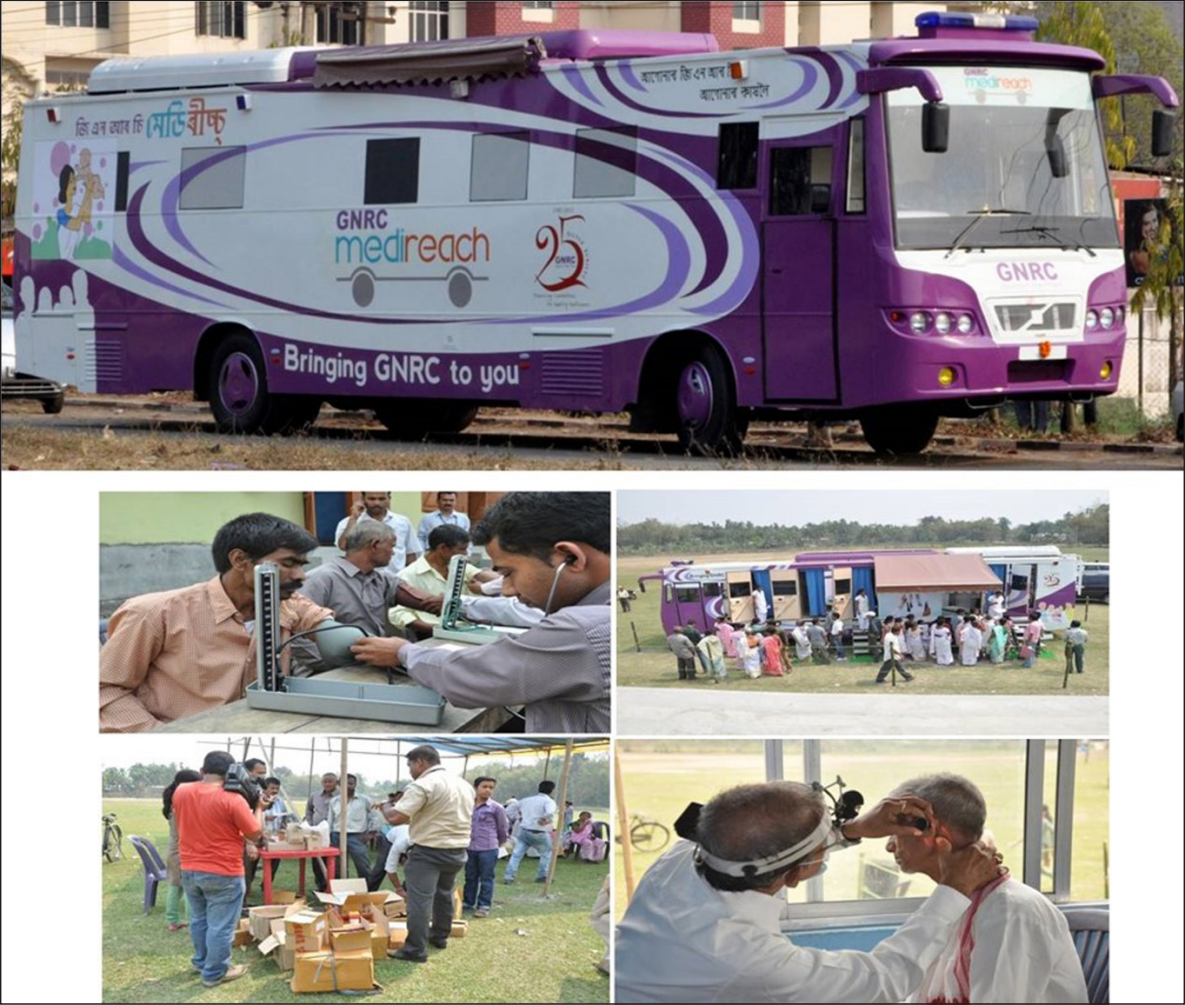
GNRC’s Medireach bus with ECGs, blood sugar, ultrasonography, X-ray, and pap-smear facility.

The integration at GNRC started in the year 2012 with a pilot outreach project called Medireach. GNRC Medireach was a mobile hospital unit which was capable of conducting ECGs, blood sugar, X-Ray, Ultrasonography, Lab, and Pap smear test facilities along with a medical team and supplies. They also collect, store, and transport blood and urine samples for other tests that are conducted at the hospital laboratories. The vehicle visited the communities, raised awareness about seeking timely care, and regular management of chronic conditions, and provided curative care to the ill. This initiative addressed the huge unmet need for easily accessible care in the communities as the patients did not have to travel long distances, loss wages, incur expenses on transport, and very often delay in care-seeking. Medireach has scaled up to cover around 15 million people in 12 districts of Assam to provide reliable and affordable diagnostic services to the community people. The initiative has increased awareness among the communities about GNRC and consequently increased the number of patients that come to GNRC for care. To date, GNRC Medireach has covered 1,339 no of trips and has scanned 276,024 patients (Figure 3).

### GNRC’s Swasthya Mitra

**Figure 4:**
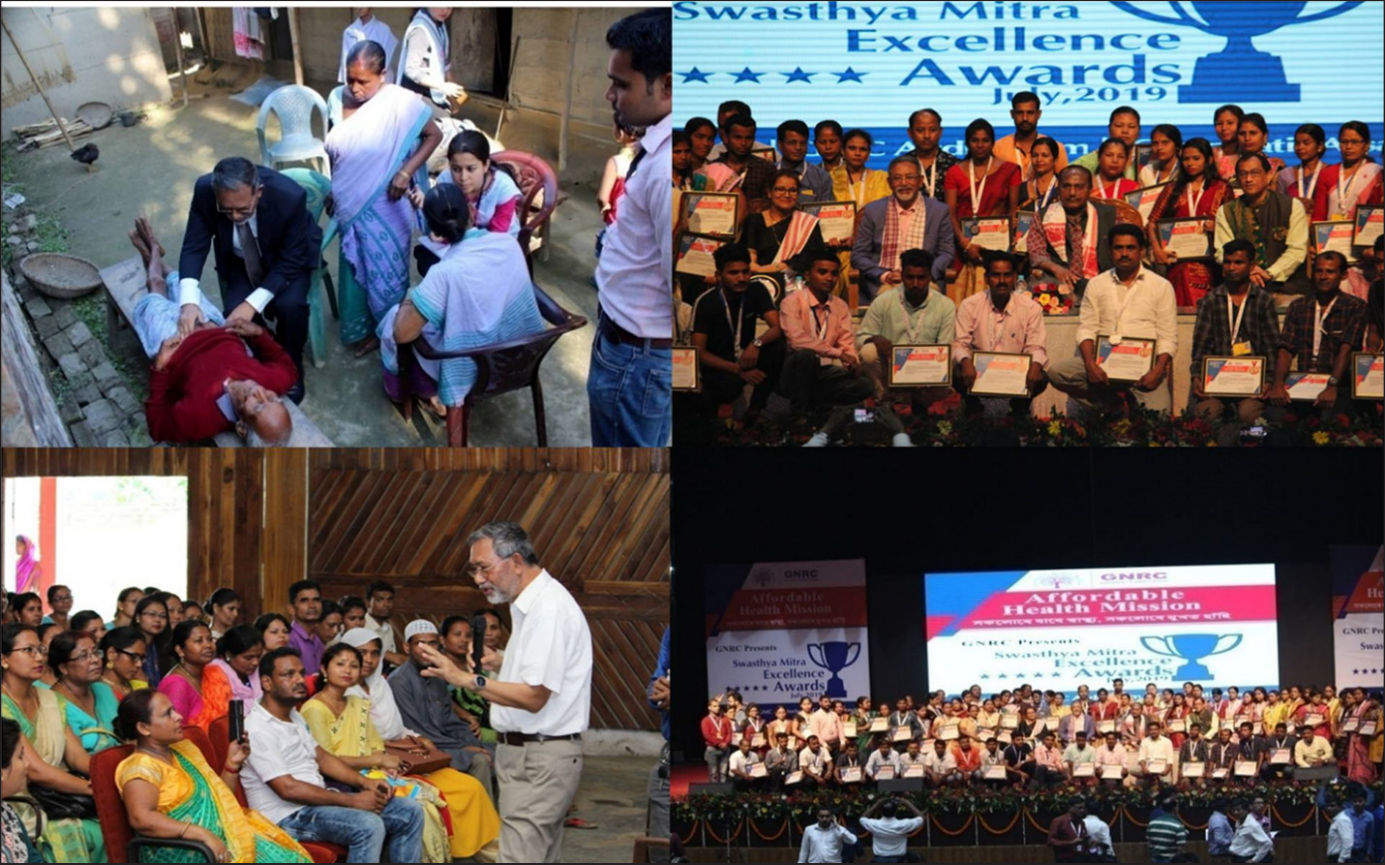
GNRC’s interacting session with the Swasthya Mitras and Community visit programme.

GNRC through its Affordable Health Mission (AHM) has formulated a unique and ambitious initiative called “Swasthya Mitra” in the year 2015. GNRC’s Swasthya Mitra is the pool of community health workers who constantly work for preventive and promotive care for Assam’s rural and semi-urban impoverished population. Most Swasthya Mitras are selected from a local community and most of them are women from the age group 25-50 years with a minimum qualification of high-school education. They are the frontline health workers who have the necessary understanding of their community, to serve as intermediaries between doctors and the community. Swasthya Mitra visits the people of rural areas and mentors them in practicing various preventive and promotive health care measures like hand washing, how to purify water before drinking, food hygiene, sanitation, road safety, dietary habits, the menace of alcohol, tobacco, & drugs and the need to avoid those. Also, the importance of taking care of lifestyle diseases like high blood pressure, diabetes, etc. is communicated during their visits. GNRC’s this primary care initiative has grown beyond Medireach into a well-planned community health worker program. Till now GNRC has trained ~20000 community health workers i.e., Swasthya Mitra (which means “A Friend for Health”) and currently 6000 active Swasthya Mitra are there who provide basic primary and preventative care for 30 million people across 30 districts in the state. Swasthya Mitra aims to identify high-risk cases of non-communicable diseases and acute illnesses and refer them to the GNRC hospitals. Each Swasthya Mitra looks after 400 households, enumerates his/her catchment, and maintains health records of each individual on existing conditions as well as at-risk indicators. While the Swasthya Mitra is similar in profile to the community health workers under India’s national ASHA program, the difference lies in her roles and responsibilities. The Swasthya Mitra’s focus is on both communicable and non-communicable diseases including other illnesses, while the ASHAs focus on pregnant women and small children. All other patients who need curative care or diagnosis are referred by the Swasthya Mitra to the GNRC Hospitals. Almost 60% of GNRC’s patients are from the communities and are referred by the Swasthya Mitra (Figure 4).

### GNRC’s Telehealth services

GNRC’s Telehealth is an innovative option by which patients can consult with GNRC specialists even from their homes. So, it is an interface between Patients and Doctors in the periphery. To date there are 6000 active walking Telehealth service providers operated through Android smartphones by Swasthya Mitras. Telehealth offers healthcare services like consultation with GNRC doctors, home delivery of advised medications, guidance to get routine investigations locally, etc. to the community at the comfort of their homes. This has immensely helped people living in remote areas and a significant amount of money and time has been saved for the people.

### Redesigning of GNRC’s Care Team

Re-designing the composition of the care team is another key innovation to GNRC’s model. GNRC’s care team mainly comprises Swasthya Mitras, laboratory technicians, nurses, general physicians, and specialists. Traditionally, in the Assam catchment enumeration and identification of high-risk cases are not done yet even by primary care teams. GNRC’s Swasthya Mitra undertake this regularly in their communities, along with maintaining population-based health records that eventually help in the prevention and progression of the disease, and finally, it facilitates timely care.

The outpatient care teams of GNRC hospitals comprise nurses and a physician. They function like a more typical primary care team; except they have greater information about their patients (through the community-based care of Swasthya Mitras). Therefore, they are better able to ensure the right referral decisions, and are assured of continuity of care by the Swasthya Mitras whether in the hospital or post-discharge. GNRC’s hospital departments are headed by specialists. A number of them are graduates with a two-year training course for specialists that was introduced to address the huge specialist shortage in the state. The complicated cases needing advanced care are overseen by the specialists, while physicians and nurses effectively manage most cases for clinical out-patient care, and by the Swasthya Mitras in the community.

### Promoting provider behavior that prioritizes quality and accountability

GNRC has designed several interventions to ensure that its providers are incentivized to prioritize quality and accountability in their services.

#### Human resource recruitment and selection

GNRC pays a significant amount of attention to the recruitment and selection of its staff to ensure better alignment with the organization’s vision. GNRC recruits early and mid-career medical graduates rather than senior doctors as senior doctors work according to their practice. But it is easier to train junior doctors and their cost is also low and it is an attraction for them because, during the formative period of their career, they need to access a large number of patients to make sure they are skilled and can get much experience. Therefore, these doctors are more ‘trainable’ to GNRC’s approach to healthcare, as well as they are more sensitive to the needs of the patients; which motivates them to provide patient-centric care.

#### Redesigning provider payments

GNRC has aligned the incentives of their clinical team with the objectives of assuring quality and accountability to their patients. GNRC pays fixed salaries to their doctors with a component of the performance bonus determined by the management based on predefined performance criteria such as compliance with clinical processes, their treatment outcomes for patients, patient’s feedback on the perceived quality of the doctor, contributions to research and publications, and the volume of patients that they treat. In the case of promotions of doctors, GNRC includes criteria such as the length of service and loyalty, quality of service, research, and contributions towards bringing down the cost of treatment. As a result, doctors do not ask patients for unnecessary “follow-up” visits, and most conditions are effectively managed by Swasthya Mitras in the community. Generally, each Swasthya Mitras are paid INR 20 (~$0.25) for their household visits and to promote the people’s health-seeking behavior which brings their basic earnings to INR 4000 (~$50) per month. For helping a patient seek appropriate care at a GNRC hospital outpatient department or from the Medireach team, and facilitating the process at a GNRC hospital for the patient, the Swasthya Mitra receives a share of the fees. A Swasthya Mitra on average, earns around INR 6000–7000 per month (~$75–87), to 50000 (~612.15) per month and which is higher than the average income in the state.

### Delivering easily accessible and affordable care

GNRC has undertaken several initiatives to make services patient-friendly, easily accessible, and affordable to address the barriers commonly faced by patients.

#### Convenience of care and transportation for patients

For the convenience of patient care, GNRC runs an OPD clinic for 12 hours a day. For OPD patients the hospital provides free guidance from Swasthya Mitra and runs free for the patients who come from satellite towns and villages which address the lack of reliable and affordable transportation for people to access care.

#### Low-cost, same-day diagnostics

As most patients lose multiple days’ wages due to repeated visits for diagnostics, laboratory reports, and medicines when they seek care, therefore GNRC has started a patient-centered initiative to run and complete all diagnostic tests within the same day (even for out-patient or primary care). All branches of GNRC hospitals are a one-stop shop with fully equipped pharmacies, laboratories, and imaging facilities. Each OPD visit is completed in one day so that patients do not incur additional and indirect expenses like transportation or accommodation when they come from far-flung areas. Because of this convenience, the volume of patients accessing diagnostic services is high which helps GNRC utilize its equipment to the maximum, often a problem with most diagnostic facilities.

#### Access to Affordable care and low unit costs for treatment

GNRC hospital provides free medical care to all accident & emergency patients for the first 24 hours after an emergency. The free facility covers ambulance services (there is a total of 59 ambulances till now and expecting to be 100 soon) nominal registration services, doctors’ consultation, many blood tests, bed charge, and oxygen, 38 types of essential medicines and consumables, CT scan (Brain), ECG, and X-rays. Apart from that those who require admission accommodation in the ICU or wards are provided free for the first 24 hrs. For OPD patients the hospital provides free guidance from Swasthya Mitra, free transport services to and fro from the hospital, and free doctor’s consultations. Use of generic medicines, procuring India-made medical supplies at cheaper costs, and use of high-end diagnostics several times a day (running for multiple deserving patients) bring investment costs down and usually help result in large savings for both hospitals and patients. High-cost investigations are performed at a nominal cost – for instance, CT Brain at USD 12, MRI Brain at USD 35, and Coronary Angiography at USD 77. Reduced cost of surgeries such as Laparoscopic Gall Bladder Surgery at USD 371, Cataract Surgery at USD 143, and Uterus Removal Surgery at USD 457, etc. In a day GNRC hospitals altogether conduct at least 50 surgeries, thus even if the first 24 hours of admission are free, the hospital is still able to make profits due to the large volume of admissions and more surgery patients. In addition, the hospital provides free bus service for the patients and their attendants regularly from locations up to 400 kilometers away, and even further, as per requirement. Apart from convenience and affordability, the specialty of the GNRC hospital campus is the environment-friendly infrastructure. To limit expenses, bamboo cladding is used extensively in the building’s exterior. That acts as a natural insulator and allows the hospital to do away with air conditioning in common areas such as lobbies and reception. This resulted in saving electricity and reducing the maintenance cost of the hospital.

#### Economic empowerment and alignment with Government health insurance schemes

GNRC undertakes innovative income-generating schemes for the poor people to address the problem of poverty, which provides guaranteed income of INR 10,000 per family every month – along with health insurance which does not come within the purview of schemes like Pradhan Mantri Jan Arogya Yojana (PM-JAY). The GNRC hospitals are empanelled under PM-JAY and Atal Amit Abhiyan (AAA) from the Government of Assam and have seen the third-highest patient volumes under this scheme in the state [29]. Because of our cost efficiencies, the PM-JAY rates are attractive to GNRC, while most other private hospitals find the rates too low to sustain.

## Discussion

Ignorance, ill health, and poverty are the biggest enemies of mankind which increase social inequality. GNRC has challenged this menace of mankind by developing a sustainable healthcare delivery model which addresses the issues of awareness, prevention, affordable treatment, and economic empowerment of the financially weaker sections of society. Through this model, the Swasthya Mitra of GNRC hospitals provide health education at the community level and create health awareness amongst the masses. There are several private sector organizations that have already developed a simpler and cheaper service that have contributed as a source of “disruptive innovators [30]. These organizations have improved the affordability, availability, or quality of care for the poor and explored the potential of these models to create more inclusive and effective health services in resource-limited settings. One such organization is the K-MET (Kisumu Medical and Educational Trust) from Kenya which was established in the year 1995 for maternal and child care. They provide training to existing providers on reproductive health, family planning, safe abortion care. They also provide care for rural communities where government services are unavailable. Apart from that they give loans to clinics and provide training to improve facilities and ensure safety and high quality of care [31]. Another organization named Dentista Do Bem from Brazil was established in the year 2002 for dental Care for youths. They provide free treatment provided by existing practitioners and they have reached 12,000 children in 27 states in Brazil in the year 2009. This model has been replicated in 6 Latin American Countries [32]. The Luoho model in Shenzhen, is one of the leading examples of integrated care system on the value of inpatient care in China. The model has suggested that it has reduced inpatient costs and length of hospital stay without sacrificing the decisive quality metrics such as 30-day readmission among the elderly population [33].

From the Indian context, the way GNRC has conceptualized its care process for the patient is an innovative approach. It provides a good example of how an organization can successfully combine patient-centered care with financial sustainability – two goals that are often perceived as contraposed to each other, especially for private sector healthcare in India. As “Affordable health mission” of GNRC hospital is in the system for the last 8 years and it has been designed in such a way that though it is low cost it has been tested and it appears to be a sustainable model as it generates a sufficient amount of revenue due to its high volume of the patient pool. The two factors that make a strong case for the sustainability of this model first, our Swasthya Mitra contributes to almost 86 percent of its revenue and 81 percent of the patient footfall by covering about half of the communities in the district and campaigning <10 percent population. Second, the market size is huge and underserved. As GNRC system has four hospitals near Guwahati which has a viable catchment area of 10 districts; Assam has 35 districts and India has 770 districts which demonstrate the size of the potential market. Given it is an economical yet private profit-making hospital, we can expect that the target consumers for the GNRC model would be the middle-class population that earns ◽2–10 lacs per year and is roughly estimated to be 20 percent of India’s population. Moreover, schemes such as PMJAY can expand the market by at least twice the size of providing insurance cover to the poorer population. To scale up our operating model, World Bank Group (WBG) funded an amount of US$150,000 in the year 2014 [34]. Moreover, USAID-SAMRIDH in the year 2021–22 also supported GNRC hospitals to scale up our number of Swasthya Mitra, Free Ambulance, and Free Transport Services to fast-track India’s response to the COVID-19 pandemic with funding of US $ 1.2 million. With the extension of SAMRIDH grant, GNRC should be able to utilize about half of its excess capacity and with a network of 7,500+ Swasthya Mitra the hospital can cover all communities of the district, reach 20 percent population, and mobilize enough patients to run GNRC at full capacity. A primary case report has also been submitted to Harvard T.H. Chan School of Public Health, Boston, Massachusetts in the year 2020 regarding GNRC’s integrated care system [35].

Accessing secondary and tertiary healthcare is particularly challenging in countries like Myanmar, Bangladesh, Africa, Nepal Bhutan, etc. Beyond the poor state of the health system itself, people face several barriers to accessing services. Most people only have access to primary healthcare services, which are not robust enough to provide care for non-communicable diseases. Presently patients from countries like Nepal, Bhutan, Bangladesh, and Myanmar come to GNRC for treatment and in near future we are also planning to set up new super specialty hospital facilities in those regions and countries where affordability is an issue.

## Conclusion

The proposed model “Affordable Health Mission” is the first innovative approach from Northeast India, Assam, which is delivering affordable, accessible and patient-centric community-based preventive, promotive, and primary, secondary, and tertiary hospital-based care. It is projected that GNRC’s “Affordable Health Mission” will help the way primary healthcare is delivered to the population of Assam by helping patients manage their health and reduce the numbers that need to be admitted to secondary and tertiary care by improving patient’s independence and well-being as well as dramatically reducing the cost to the overall health system. Therefore, GNRC’s care delivery model may lead to meaningful benefits for patients and help hospitals best serve the health needs of the communities of Assam and other parts of North-eastern India.

## References

[1] Syed MA, Al Mujalli H, Kiely CM. Development of a model to deliver primary health care in Qatar. Integrated Healthcare Journal. 2020; 2(1): e000040. 37441307 10.1136/ihj-2020-000040 PMC10327457

[2] Nongkynrih B, Patro BK, Pandav CS. Current status of communicable and non-communicable diseases in India. Journal of the Association of Physicians of India. 2004; 52: 118–123. 15656045

[3] Enthoven AC. Integrated delivery systems: the cure for fragmentation. American Journal of Managed Care. 2009; 15(12): S284–290. 20088632

[4] Frandsen BR, Joynt KE, Rebitzer JB, Jha AK. Care fragmentation, quality, and costs among chronically ill patients. The American Jounal of Managed Care. 2015; 21(5): 355–362. 26167702

[5] Stange KC. The problem of fragmentation and the need for integrative solutions. The Annals of Family Medicine. 2009; 7(2): 100–103. DOI: 10.1370/afm.971 19273863 PMC2653966

[6] Maruthappu M, Hasan A, Zeltner T. Enablers and barriers in implementing integrated care. Health Systems & Reform. 2015; 1(4): 250–256. DOI: 10.1080/23288604.2015.1077301 31519094

[7] Brown CL, Menec V. Integrated care approaches used for transitions from hospital to community care: A scoping review. Canadian Journal on Aging. 2018; 37(2): 145–170. DOI: 10.1017/S0714980818000065 29631639

[8] Druetz T. Integrated primary health care in low-and middle-income countries: a double challenge. BMC medical ethics. 2018; 19(1): 89–96. DOI: 10.1186/s12910-018-0288-z 29945623 PMC6020002

[9] Tan KB, Earn Lee C. Integration of primary care with hospital services for sustainable universal health coverage in Singapore. Health Systems & Reform. 2019; 5(1): 18–23. DOI: 10.1080/23288604.2018.1543830 30924743

[10] Pati MK, Swaroop N, Kar A, Aggarwal P, Jayanna K, Van Damme W. A narrative review of gaps in the provision of integrated care for non-communicable diseases in India. Public Health Reviews. 2020; 41(1): 1–6. DOI: 10.1186/s40985-020-00128-3 32435518 PMC7222468

[11] Government of India. Key indicators of social consumption in India: Education. National Sample Survey 71st Round; 2015.

[12] Choudhury S. Health scenario of Assam: A study. International Journal of Advance Research, Ideas and Innovations in Technology. 2018; 4(4): 200–203.

[13] Chandramouli C, Registrar General of India, Census of India. Ministry of Home Affairs, Government of India: New Delhi; 2011.

[14] Assam – Poverty, growth, and inequality. India state briefs. Washington, DC: World Bank Group; 2017. http://documents.worldbank.org/curated/en/545361504000062662.

[15] Sample Registration System statistical report. New Delhi: Government of India; 2017.

[16] Indian Council of Medical Research, Public Health Foundation of India, and Institute of Health Metrics and Evaluation, India: Health of the nation’s states – the Indian state-level disease burden initiative. New Delhi: ICMR, PHFI and IHME; 2017.

[17] Sheikh K, Saligram PS, Hort K. What explains regulatory failure? Analysing the architecture of health care regulation in two Indian states. Health Policy Plan. 2015; 30(1): 39–55. DOI: 10.1093/heapol/czt095 24342742

[18] Chokshi M, Patil B, Khanna R, Neogi SB, Sharma J, Paul VK, Zodpey S. Health systems in India. Journal of Perinatology. 2016; 36(3): S9–12. DOI: 10.1038/jp.2016.184 27924110 PMC5144115

[19] Alam K, Mahal A. The economic burden of angina on households in South Asia. BMC Public Health. 2014; 14(1): 179. DOI: 10.1186/1471-2458-14-179 24548585 PMC3930925

[20] Levesque JF, Haddad S, Narayana D, Fournier P. Outpatient care utilization in urban Kerala, India. Health Policy Plan. 2006; 21(4):289–301. DOI: 10.1093/heapol/czl013 16790454

[21] Kastor A, Mohanty SK. Disease and age pattern of hospitalisation and associated costs in India: 1995–2014. BMJ Open. 2018; 8(1): e016990. DOI: 10.1136/bmjopen-2017-016990 PMC5786114 29371266

[22] Peters DH. Better health systems for India’s poor: findings, analysis, and options. Washington, DC: World Bank Publications; 2002. DOI: 10.1596/0-8213-5029-3

[23] Mohanty SK, Kastor A. Out-of-pocket expenditure and catastrophic health spending on maternal care in public and private health centres in India: a comparative study of pre and post national health mission period. Health Economics Review. 2017; 7(1): 31. DOI: 10.1186/s13561-017-0167-1 28921477 PMC5603466

[24] Selvaraj S, Karan AK. Why publicly-financed health insurance schemes are ineffective in providing financial risk protection. Economic and Political Weekly. 2012; 47(11): 60–68.

[25] Ghosh S. Publicly financed health insurance for the poor. Understanding RSBY in Maharastra. Economic and Political Weekly. 2014; 49(43): 93–99.

[26] Joe W, Perkins JM, Kumar S, Rajpal S, Subramanian SV. Institutional delivery in India, 2004 –14: unravelling the equity-enhancing contributions of the public sector. Health Policy Plan. 2018; 33(5): 645–653. DOI: 10.1093/heapol/czy029 29659831

[27] International Finance Corporation. Landscape of inclusive business models of healthcare in India: business model innovations. World Bank; 2017.

[28] Reshmi B, Unnikrishnan B, Rajwar E, Parsekar, SS, Vijayamma R, Venkatesh BT. Impact of public-funded health insurances in India on health care utilisation and financial risk protection: a systematic review. BMJ open. 2021; 11(12): e050077. DOI: 10.1136/bmjopen-2021-050077 PMC8704974 34937714

[29] National Health Authority. www.pmjay.nic.in; 2019.

[30] Hwang J. Christensen CM: Disruptive Innovation In Health Care Delivery: A Framework For Business-Model Innovation. Health Affairs. 2008; 27: 1329–1335. DOI: 10.1377/hlthaff.27.5.1329 18780919

[31] Montagu D, Prata N, Campbell MM, Walsh J, Orero S. Kenya: Reaching the poor through the private sector – A network Model for expanding access to reproductive health services; 2005.

[32] Bhattacharyya O, Khor S, McGahan A, Dunne D, Daar AS, Singer PA. Innovative health service delivery models in low- and middle-income countries-what can we learn from the private sector? Health Research Policy System. 2010; 8(1): 1–11. DOI: 10.1186/1478-4505-8-24 PMC3236300 20630108

[33] Ye Z, Jiang Y. The impact of a pilot integrated care model on the quality and costs of inpatient care among chinese elderly: a difference-in-difference analysis of repeated cross-sectional data. Cost Effectiveness and Resource Allocation. 2022: 20(1): 28. DOI: 10.1186/s12962-022-00361-4 35752860 PMC9233857

[34] World Bank Group sanctions $150,000 grant to GNRC. The Hindu; 2014.

[35] Kalita A. Delivering hospital-led integrated care: innovations by GNRC Hospitals, Assam; 2020.

